# Israeli Spotted Fever *Rickettsia* in Sicilian *Rhipicephalus sanguineus* Ticks

**DOI:** 10.3201/eid0907.030109

**Published:** 2003-07

**Authors:** Giovanni M. Giammanco, Serafino Mansueto, Pietro Ammatuna, Giustina Vitale

**Affiliations:** * Università di Palermo, Palermo, Italy

**To the Editor:** Mediterranean spotted fever (MSF) is endemic in Italy, where it is a reportable disease. From 1992 to 1998, the Italian Ministry of Health was notified of approximately 8,500 cases of human rickettsioses presumed to be MSF. MSF occurs more commonly in some central (Lazio) and southern (Sardinia, Sicily, and Calabria) regions (*1*,*2*); in 1998, an average of 8.8 cases occurred for every 100,000 persons in Sicily, compared with the national average of 1.6 cases per 100,000 persons. *Rickettsia conorii* has been thought to be the only pathogenic *Rickettsia* of the spotted fever group in Sicily (*3*,*4*) or the western Mediterranean area. Recently, three different spotted fever group rickettsiae, including *R. helvetica*, were detected in *Ixodes ricinus* ticks from central and northern Italy. This finding suggests that bacteria other than *R. conorii* are involved in rickettsial diseases in Italy (*5*).

To investigate whether unusual tick-transmitted rickettsiae are also present in Sicily, we used molecular-sequence–based identification techniques to study two strains isolated from the hemolymph of *Rhipicephalus sanguineus* ticks collected in 1990 in western Sicily. These isolates had been previously identified by serologic tests as belonging to the spotted fever group rickettsiae. We obtained bacterial DNA and performed polymerase chain reaction (PCR) for *ompA* gene and restriction analysis under conditions previously described by Roux et al. (*6*). Our observation of a peculiar *Pst*I profile allowed a presumptive identification of one of the two tick isolates as belonging to the Israeli spotted fever rickettsiae, while the other showed a restriction profile corresponding to that of *R. conorii* strain Seven. To confirm the identification of the Israeli spotted fever *Rickettsia* isolate, we sequenced the PCR-amplified fragment of *omp*A gene (MWG-Biotech AG, Ebersberg, Germany) and aligned sequence data with homologous sequences of reference strains of the spotted fever group rickettsiae retrieved from the GenBank database. Sequence analysis showed 100% similarity with the homologous sequence of Israeli spotted fever *Rickettsia* reference strain ISTT CDC1 (GenBank accession no. U43797). The Israeli spotted fever *Rickettsia* belongs to the *R. conorii* complex (*7*,*8*) and was first isolated in 1974 from ticks and humans. Initially, Israeli spotted fever rickettsiae distribution appeared to be restricted to Israel (*9*), but more recently the organism has also been isolated from patients with MSF in Portugal (*10*). Our finding of Israeli spotted fever *Rickettsia* infection in a *R. sanguineus* tick, the main vector for MSF in Sicily, also suggests that the geographic distribution of Israeli spotted fever might be wider than previously thought, including not only Israel and the Iberian Peninsula but also Italy.

Molecular analysis of spotted fever group *Rickettsia* isolates from Sicilian MSF patients is under way to verify this hypothesis. Because initial signs and symptoms of Israeli spotted fever are particularly uncharacteristic, awareness of the presence of Israeli spotted fever *Rickettsia* in our geographic area may hasten provision of the appropriate treatment. The Sicilian *omp*A gene sequence described in this study has been deposited in the GenBank database (accession no. AY197565).
